# Exploring the mechanism of fraxetin against acute myeloid leukemia through cell experiments and network pharmacology

**DOI:** 10.1186/s12906-024-04529-8

**Published:** 2024-06-10

**Authors:** Tingting Fang, Lanqin Liu, Wenjun Liu

**Affiliations:** Department of Pediatrics (Children Hematological Oncology), Children Hematological Oncology and Birth Defects Laboratory, Sichuan Clinical Research Center for Birth Defects, The Affiliated Hospital of Southwest Medical University, Luzhou, Sichuan 646000 China

**Keywords:** Fraxetin, Acute myeloid leukemia, Cell proliferation and apoptosis, Network pharmacology

## Abstract

**Objective:**

Previous studies have shown that fraxetin has antitumor activity in a variety of tumors, but its role in acute myeloid leukemia (AML) remains unclear. In this study, we aimed to evaluate the anti-AML effect of fraxetin through cell experiments and network pharmacology analysis.

**Methods:**

The inhibitory and apoptotic effects of fraxetin on AML cells were determined by CCK-8 and flow cytometry experiments. Potential targets of fraxetin and AML-related targets were screened using public databases. PPI network, GO functional enrichment and KEGG pathway enrichment analyses were performed to predict the hub targets and signaling pathways by which fraxetin alleviates AML. Molecular docking was used to determine the fraxetin binding sites on hub targets. Using the GEPIA database, the expression of hub targets was analyzed in relation to the overall survival of AML patients.

**Results:**

Cell experiments showed that fraxetin inhibits AML cell proliferation and induces apoptosis. To explore the potential mechanism of fraxetin, 29 shared targets of fraxetin and AML were obtained through screening online public databases. Among them, AKT1, TNF, SRC, etc., are related to AML cell apoptosis. The expression levels of SRC, NOS3, VAV1, LYN, and PTGS1 were associated with the overall survival of AML patients (p value < 0.05). The enrichment analysis results identified the main pathways, namely, focal adhesion and the PI3K-AKT signaling pathway, that affected the proliferation and apoptosis of AML cells. The analysis of hub targets of the PPI network showed that AKT1, TNF, CTNNB1, etc., were hub targets, which were related to the proliferation and apoptosis of AML cells. The results of molecular docking showed that the hub targets had good binding with fraxetin.

**Conclusion:**

Fraxetin may inhibit AML cell proliferation and induce AML cell apoptosis through multiple targets, such as AKT1, SRC, and EGFR, and multiple pathways, such as focal adhesion and the PI3K-AKT signaling pathway.

**Supplementary Information:**

The online version contains supplementary material available at 10.1186/s12906-024-04529-8.

## Introduction

Acute myeloid leukemia (AML) is a heterogeneous hematological tumor whose pathogenesis involves the malignant proliferation of leukemia cells in the bone marrow, which leads to normal hematopoietic dysfunction in the bone marrow, and its clinical manifestations are bone marrow suppression or the extensive infiltration of various organs in the patient [1]. AML is the most common type of leukemia in adults, accounting for 15–20% of childhood leukemia cases, and its 5-year survival rate is only approximately 30% in adults and 46–69% in children [2]. A combination of chemotherapy, radiotherapy and hematopoietic stem cell transplantation is the main treatment for AML. However, the emergence of multidrug resistance significantly hinders the efficacy of chemotherapy and leads to poor prognosis [3]. In addition, adverse reactions to chemotherapy drugs and radiotherapy greatly reduce the quality of life of patients. Therefore, finding new anti-AML drugs and potential therapeutic targets is imperative to improve therapeutic efficacy against AML.

Traditional herbal medicine have the characteristics of multiple targets, multiple pathways, low price, and few side effects and have been widely accepted as a supplementary therapy for cancer [4]. Fraxetin (Supplementary file Figure S1), a natural coumarin compound, is an active ingredient in the herbal medicine Cortex Fraxini [5]. Modern pharmacological studies have shown that fraxetin can exert various anticancer, anti-inflammatory and antibacterial effects and has important research value and potential application prospects [6]. In recent years, an increasing number of studies have reported the anticancer effect of fraxetin on a variety of tumors, such as pancreatic cancer [7], breast cancer [8], and colon cancer tumors [9]. However, whether fraxetin has an anticancer effect in AML has not been determined.

**Fig. 1:**
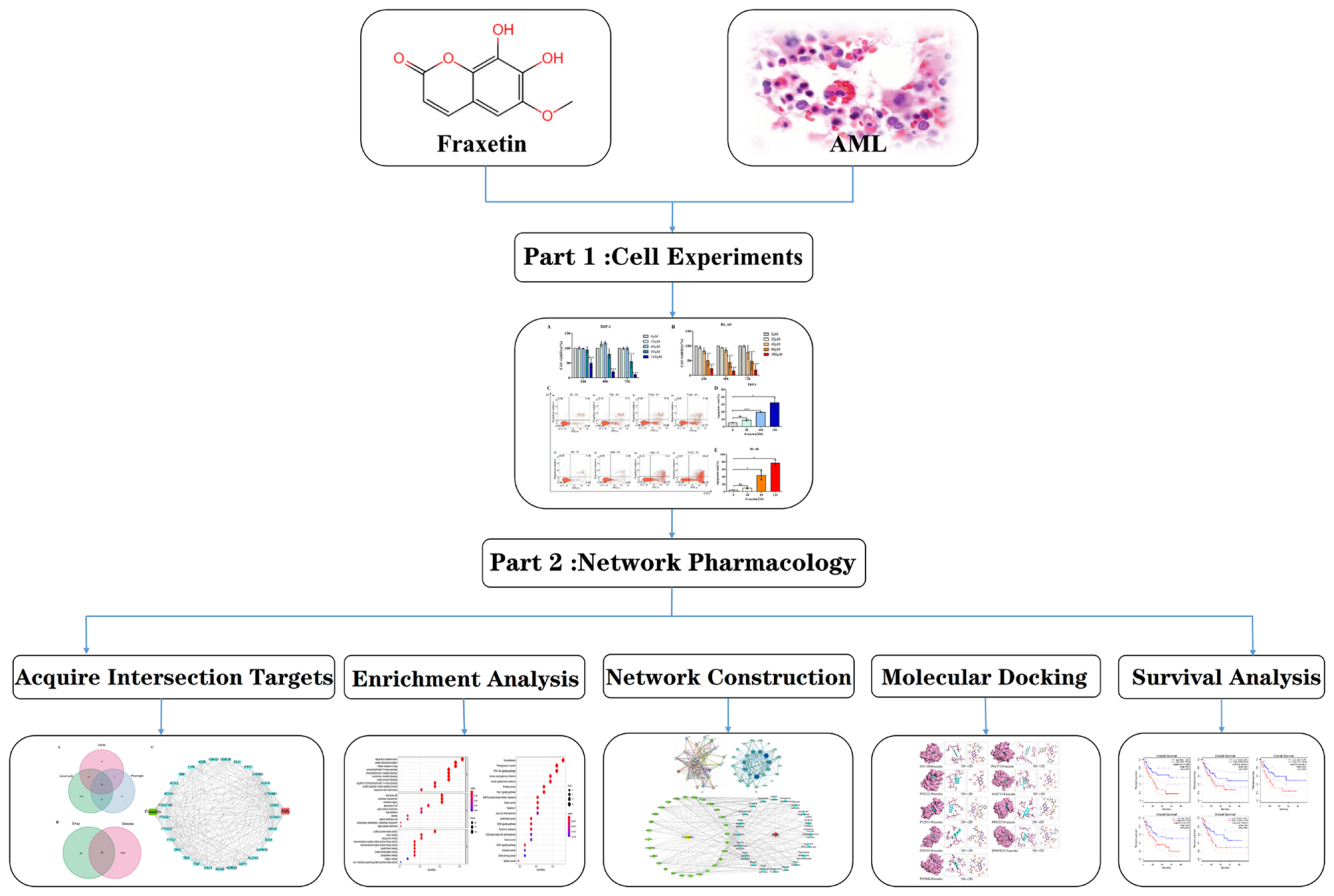
The workflow diagram

With the development of bioinformatics, data science, systems biology, etc., the research strategy to explore the interactions between drugs and diseases has gradually shifted from isolated research to systematic analysis [10]. Network pharmacology has changed the previous model of drug development to “one disease, one drug, multiple targets” [11]. Molecular docking technology is used to analyze the binding capacity and mode between active ingredients and targets, identify the active ingredients that may play a pharmacological role, and provide a reference for basic experimental research; molecular docking has been widely used in preliminary research on drug development in combination with network pharmacology [12, 13]. Therefore, in this study, we used fraxetin as the treatment and explored the effects of fraxetin on AML cells through cell experiments and through network pharmacology methods from the multiple target and pathway perspective; the results were verified by molecular docking technology to reveal the potential mechanism of action of fraxetin against AML and provide a theoretical basis for the clinical application of fraxetin. A diagram of the workflow is shown in Fig. 1.

## Materials and methods

### Experimental Verification in vitro

#### Cells and reagents

The human acute myeloid leukemia THP-1 and HL-60 cell lines were obtained from the Chinese Academy of Sciences Cell Bank (Shanghai, China). The cell lines were cultivated in Roswell Park Memorial Institute (RPMI)-1640 medium with 10% fetal bovine serum (FBS) and 1% penicillin and streptomycin at 37 °C in a humidified atmosphere of 95% air and 5% CO2. Fraxetin (> 98%) was purchased from Shanghai Duo Xi Biological Company of China. The 100 mM stock solution was made in dimethyl sulfoxide (DMSO) purchased from Beijing Solarbio Science and Technology Company of China.

#### Cell culture and viability analysis

Cell viability was evaluated using a cell counting kit-8 (CCK-8) assay (Beyotime, Shanghai, China) according to the method described by HUANG et al. [14]. THP-1 and HL-60 cells were seeded in 96-well plates, and both were treated with 0, 20, 40, 80, and 160 µM concentrations of fraxetin for 24, 48 and 72 h.

#### Apoptosis assay

Apoptosis was detected by flow cytometry with a FITC Annexin V apoptosis detection kit (BD Biosciences Pharmingen, San Diego, CA). In brief, 6.0 × 10^5^ cells were seeded in 6-well plates and treated with different experimental concentrations of fraxetin for 48 h. Then, the following steps, similar to Guo’s methods, were performed [15]. The state of 1.0 × 10^5^ out of 6.0 × 10^5^ cells in each sample was analyzed by flow cytometry (BD FACSVerse flow cytometer, San Diego, CA).

#### Statistical analysis

The results were statistically analyzed using SPSS 20.0. The experimental data are presented as the mean ± SD. ANOVA was used when the data had a normal distribution; if the result of the test for homogeneity of variance was *p* > 0.05, LSD was used to compare the two sets of data; if *p* < 0.05, the approximate F test Welch method was used for ANOVA, and then Dunnett’s T3 method was used for comparison. A nonparametric test was used when the data did not conform to a normal distribution. The experiments were repeated three times, and *p* < 0.05 indicated that the difference was statistically significant.

### Network Pharmacology Analysis

#### Collection of potential targets of Fraxetin in AML and compound-disease-target (C-D-T) network construction

The molecular structure (sdf format) of fraxetin was obtained from the PubChem database (https://pubchem.ncbi.nlm.nih.gov/*).* The potential targets of fraxetin were screened from the following databases: the Traditional Chinese Medicine Systems Pharmacology (TCMSP) database (https://www.tcmspe.com/*)*, PharmMapper (http://lilab.ecust.edu.cn/pharmmapper/*)* and the Swiss Target Prediction database (http://www.swisstargetprediction.ch/*).* Human AML-related targets were sorted from the following databases: the GeneCards database (https://www.genecards.org/*)*, the Online Mendelian Inheritance in Man database (OMIM, http://www.omim.org/*)* and the Pharmacogenetics and Pharmacogenomics Knowledge Base database (Pharmgkb, https://www.pharmgkb.org/*).* The keywords “acute myeloid leukemia” was applied as a search term, and duplicate target genes were removed. Furthermore, the UniProt database (https://www.uniprot.org/*)* was used to obtain the official names of targets, with the species defined as “*Homo sapiens*”, and then a diagram was drawn to analyze the overlapping targets to identify potential targets of fraxetin in AML. Finally, Cytoscape software (version 3.8.0) was used to construct the Compound-Disease-Target (C-D-T) network.

#### PPI Network Construction and Hub Target Screening

The shared targets were imported into the STRING database (https://stringdb.org/) to construct the PPI network, with the species limited to “*Homo sapiens*” and a confidence score > 0.4. The TSV format file, which was downloaded from the STRING database, was imported into Cytoscape to visualize and analyze the complex relationships between overlapping targets. The key topological parameters (degree) were used to characterize the most important nodes in the network, and higher quantitative values of topological parameters indicated a greater importance of the node [16]; hub targets were analyzed according to degree.

#### Enrichment analysis and compound-disease-target-pathway (C-D-T-P) network construction

To further understand the specific roles of the shared targets in gene function and related signaling pathways, Gene Ontology (GO) enrichment and Kyoto Encyclopedia of Genes and Genomes (KEGG) pathway enrichment analyses were conducted by using R software and the cluster profile, with only functional annotations with enrichment q-value < 0.05 used as the criterion. The “C-D-T-P” network was visualized using Cytoscape software.

### Molecular Docking

To further understand the binding mode and affinity of fraxetin to hub targets, the 2D structure of fraxetin was exported from the PubChem database, optimized by ChemBio 3D software and saved in SDF format. The 3D hub target structures were retrieved from the RCSB PDB database (http://www.rcsb.org/) and saved in PDB format using PyMOL software to remove the water molecules and the original ligands of the protein receptors. Then, the fraxetin and protein receptors were imported into AutoDock Vina software for docking, and the docking results and the binding energy values were obtained. The locations with the lowest binding energy were utilized for plotting. The 3D images plotted using PyMOL presented the interactions between the receptor protein and the ligand. Ligplot software was used for the analysis of 2D molecular docking images.

### Survival analysis in relation to Shared targets

Analysis of the overall survival in relation to 29 shared targets was performed using the GEPIA (http://gepia.cancer-pku.cn/index.html) database. A p value < 0.05 was used as a unified screening criterion.

## Results

### Fraxetin inhibits the proliferation of AML cells

**Fig. 2:**
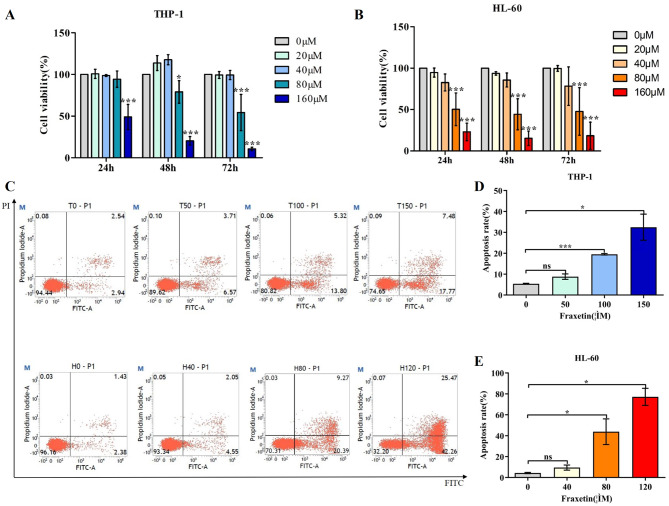
Fraxetin inhibits proliferation and induces apoptosis of THP-1 and HL-60 cells. (**A**). The viability of THP-1 cells. (**B**). The viability of HL-60 cells. (**C**). With the increase of the concentration of Fraxetin, the apoptosis rate of THP-1 and HL-60 cells also increased. (**D**) and (**E**). Comparison of apoptosis ratio of THP-1, HL-60 cells under different concentrations of Fraxetin with blank control group. (*p < 0.05, **p< 0.01,***p< 0.001, ns means no statistical significance)

To explore the effect of fraxetin on AML cell proliferation, CCK-8 experiments were performed. The results of CCK-8 experiments showed that AML cell viability was inhibited at different time points as the concentration of fraxetin increased (Fig. 2A-B). The viability of THP-1 cells was clearly inhibited after 48 and 72 h, while the viability of HL-60 cells changed significantly 24, 48 and 72 h after fraxetin treatment. Therefore, we chose 48 h as the experimental time point. Furthermore, as THP-1 cells and HL-60 cells were below 50% cell viability at concentrations of 115.1 µM and 72.3 µM, we selected 50 µM, 100 µM, 150 µM and 40 µM, 80 µM, 120 µM as the experimental concentrations for flow cytometry.

### Fraxetin induces the apoptosis of AML cells

In view of the role of fraxetin in inhibiting the proliferation of AML cells, the effect of Annexin V-FITC/PI on apoptosis in AML cells was investigated. Compared with that of the blank control group, the apoptosis rate of THP-1 and HL-60 cells gradually increased with increasing concentrations of fraxetin in the experimental group (Fig. 2C-E). The results showed that fraxetin can induce the apoptosis of AML cells.

### Acquisition of potential targets of Fraxetin in AML and the C-D-T network

**Fig. 3:**
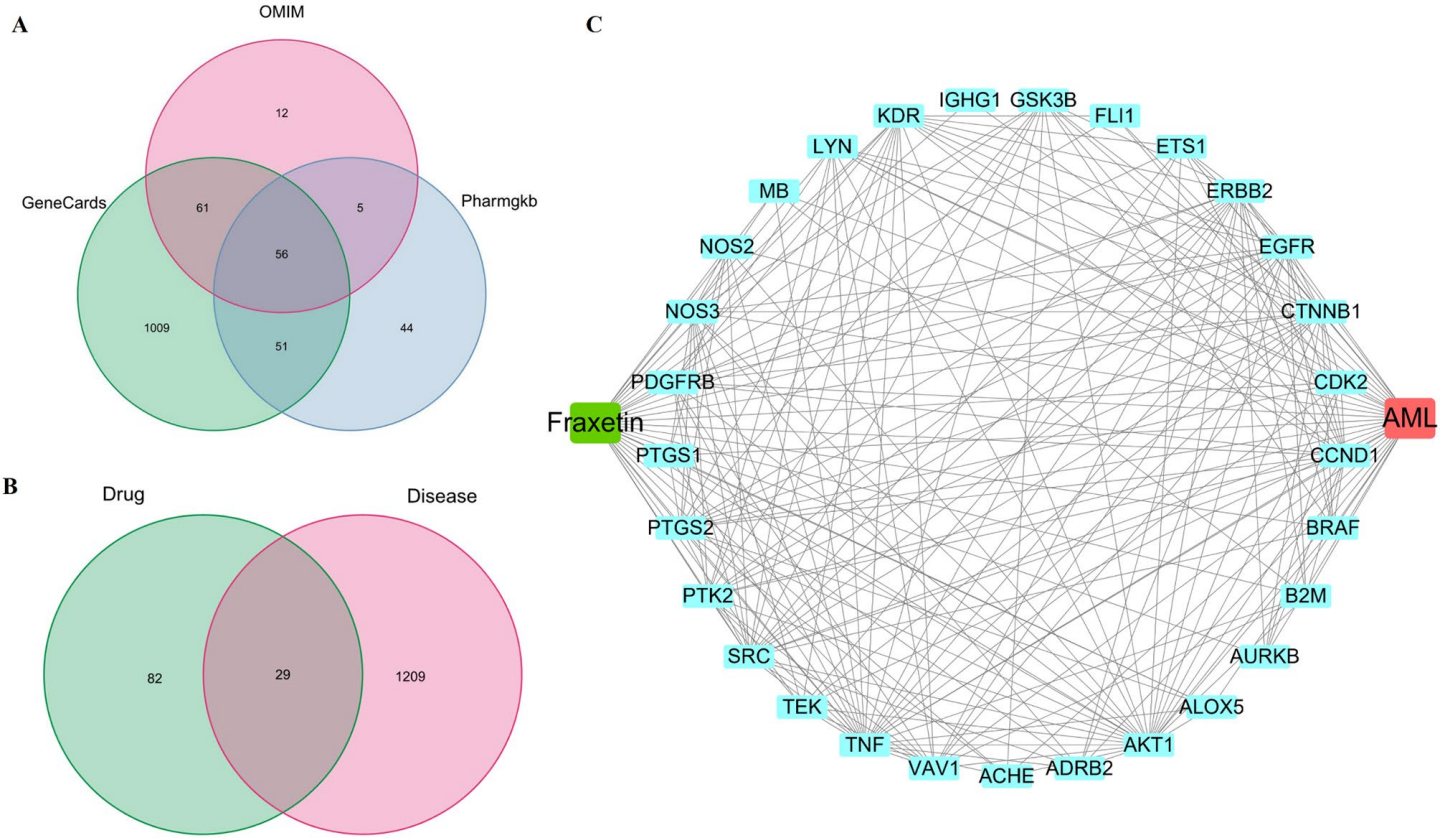
Potential targets related to Fraxetin against AML. (**A**). Veen diagram of AML-related target interactions. (**B**). Veen diagram of target intersections of Fraxetin and AML. (**C**). C-D-T network: the green module represents Fraxetin, red module represents AML, blue modules represent overlapping targets

Based on the TCMSP, PharmMapper and Swiss Target Prediction databases, a total of 111 potential targets were identified for fraxetin. AML-related gene targets were obtained from the GeneCards, OMIM and PharmGKB databases, and a total of 1238 gene targets were identified (Fig. 3A). The 111 targets of fraxetin were compared with the 1238 AML-related gene targets. Twenty-nine shared targets were considered potential candidate targets of fraxetin in AML (Fig. 3B). Cytoscape software was used to construct the C-D-T network, and the network was composed of 31 nodes and 214 edges (Fig. 3C).

### PPI network construction and hub target screening

PPI network analysis is helpful for studying the molecular mechanism of diseases and discovering new drug targets from a systematic perspective. Using the STRING database, the PPI network was constructed, and the final network contained 28 overlapping targets (nodes) and 78 interactions (edges) (Supplementary file Figure S2A). Then, according to degree, the hub targets of fraxetin in AML were obtained by Cytoscape software (Supplementary file Figure S2B). The larger and darker the node, the more connections the target had with neighboring nodes, and the more important the target; the targets included AKT1 (degree = 24), TNF (degree = 24), CTNNB1 (degree = 19), ERBB2 (degree = 18), SRC (degree = 18), EGFR (degree = 17), CCND1 (degree = 17), PTGS2 (degree = 15), and KDR (degree = 15). These targets occupied an important position in the PPI network, suggesting that they may be hub targets for the anti-AML effect of fraxetin.

### Enrichment analysis and C-D-T-P network construction

**Fig. 4:**
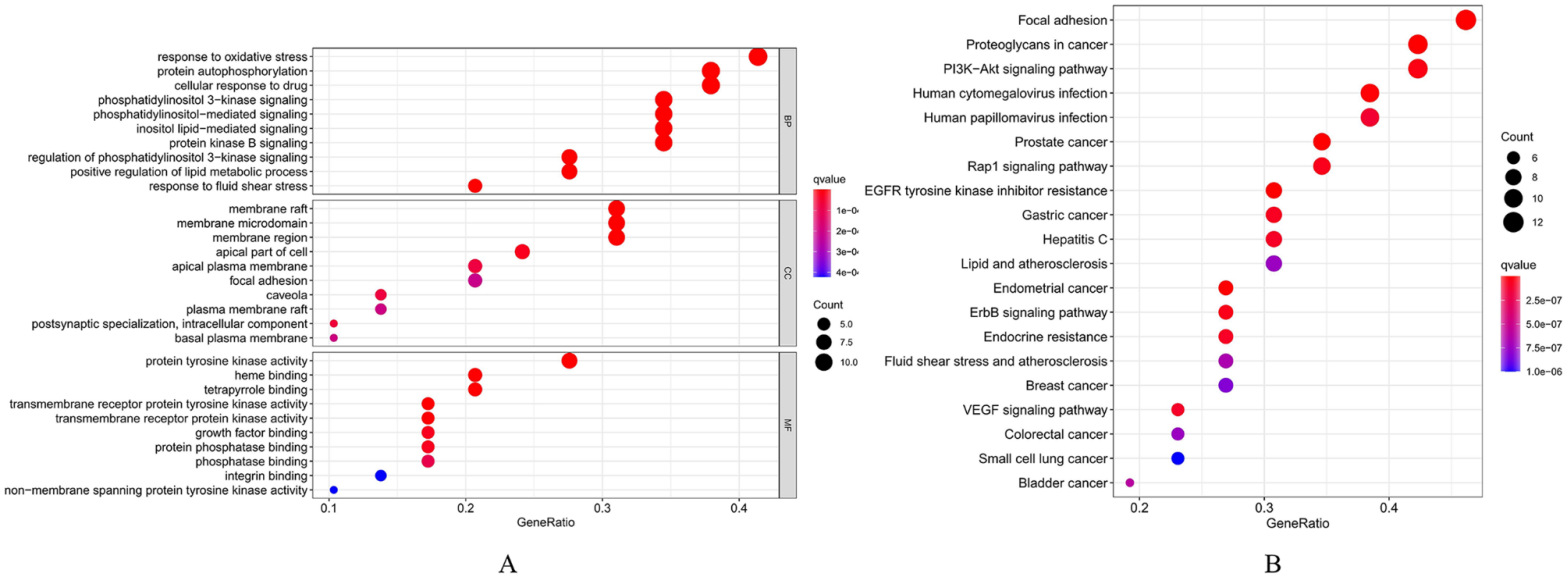
Enrichment analysis. (**A**). The Gene Ontology Enrichment analysis: the larger the plot, the greater the number of enriched targets, the smaller the q-value, the darker the plot color. (**B**). The KEGG pathway enrichment analysis: the top 20 significantly enriched pathways

**Fig. 5:**
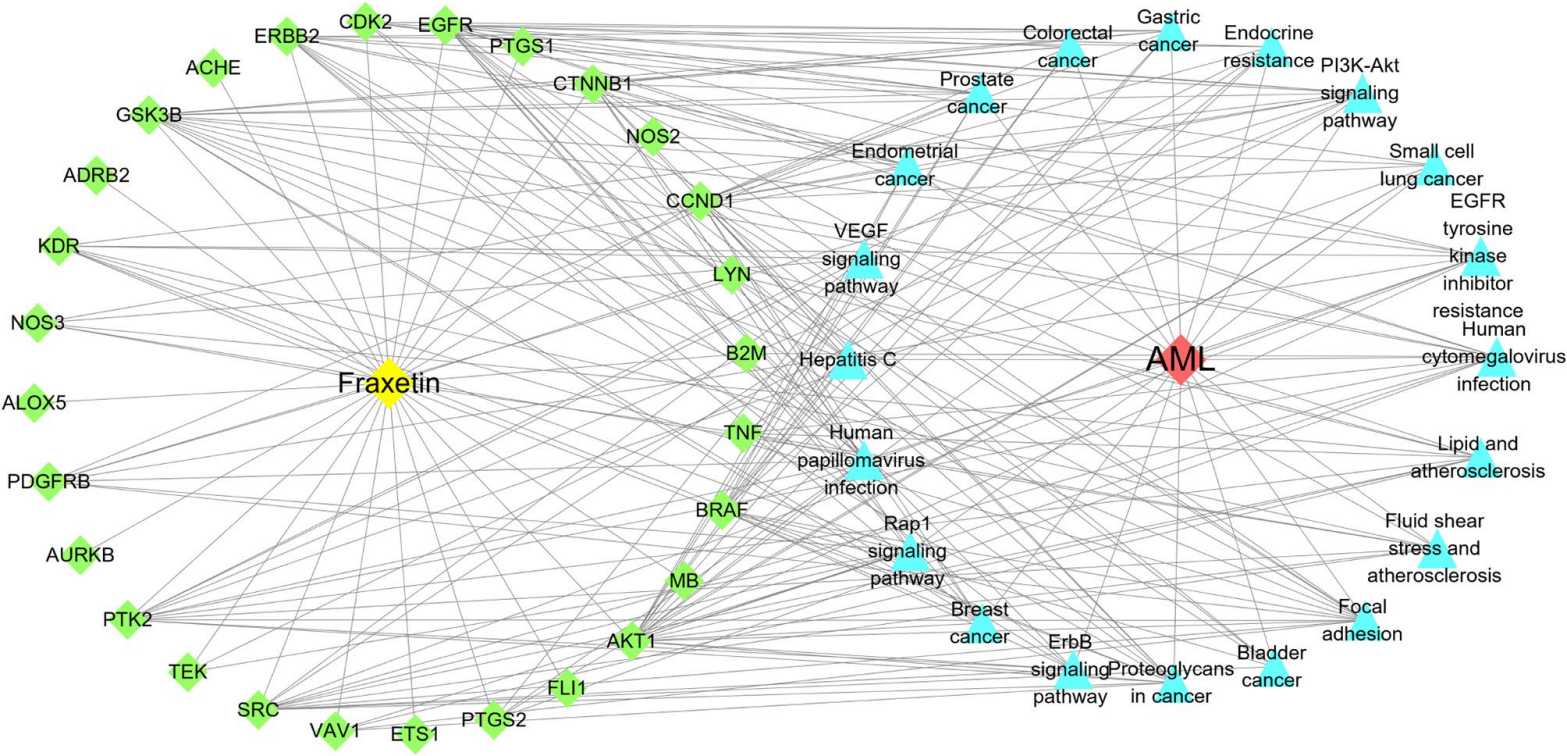
The compound-disease-target-pathway (C-D-T-P) network. The yellow module means Fraxetin, red module means AML, green modules mean overlapping targets, blue modules mean signaling pathways

To investigate how fraxetin affects AML through the hub targets, we analyzed the 29 shared targets via GO and KEGG pathway analyses by using R software. The results of GO enrichment analysis showed that the biological processes (BPs) included response to oxidative stress (GO: 0006979), protein autophosphorylation (GO: 0046777) and cellular response to drugs (GO: 0035690). The cellular components (CCs) included membrane rafts (GO: 0045121), membrane microdomains (GO: 0098857) and membrane regions (GO: 0098589). The molecular functions (MFs) included protein tyrosine kinase activity (GO: 0004713), heme binding (GO: 0020037), and tetrapyrrole binding (GO: 0046906). The GO function enrichment entries were sorted according to the q value, and the bubble map was drawn by selecting the top 10 BPs, CCs and MFs. The bubble size represents the number of enriched targets, and a larger the bubble represents more enriched targets (Fig. 4A). Thus, we hypothesized that fraxetin can exert anti-AML effects by targeting various biological functions, such as the oxidative stress response, protein autophosphorylation, tyrosine kinase activity, and cellular response to drugs. The results of KEGG enrichment pathway analysis showed that signaling pathways such as focal adhesion and the PI3K/AKT signaling pathway were the main pathways by which fraxetin impacted AML (Table 1); KEGG results were also sorted by q value (Fig. 4B). Then, the compounds, diseases, and 20 signaling pathways were imported into Cytoscape software to construct the C-D-T-P network by which fraxetin exerted anti-AML effects (Fig. 5). The results showed that fraxetin can affect multiple targets and multiple pathways in AML.

**Table 1:** Top 20 KEGG signaling pathway annotation analysis

Number	Signaling pathway	q value
hsa04510	Focal adhesion	1.53E-11
hsa05205	Proteoglycans in cancer	2.13E-10
hsa04151	PI3K-Akt signaling pathway	3.23E-08
hsa05165	Human papillomavirus infection	1.34E-07
hsa05163	Human cytomegalovirus infection	7.49E-09
hsa05215	Prostate cancer	2.13E-10
hsa04015	Rap1 signaling pathway	5.93E-08
hsa01521	EGFR tyrosine kinase inhibitor resistance	1.09E-09
hsa05226	Gastric cancer	7.25E-08
hsa05160	Hepatitis C	9.12E-08
hsa05417	Lipid and atherosclerosis	7.43E-07
hsa04012	ErbB signaling pathway	4.34E-08
hsa05213	Endometrial cancer	4.47E-09
hsa01522	Endocrine resistance	8.62E-08
hsa05418	Fluid shear stress and atherosclerosis	6.72E-07
hsa05224	Breast cancer	8.30E-07
hsa04370	VEGF signaling pathway	1.04E-07
hsa05210	Colorectal cancer	7.43E-07
hsa05222	Small cell lung cancer	1.00E-06
hsa05219	Bladder cancer	6.05E-07

### Molecular docking

**Fig. 6:**
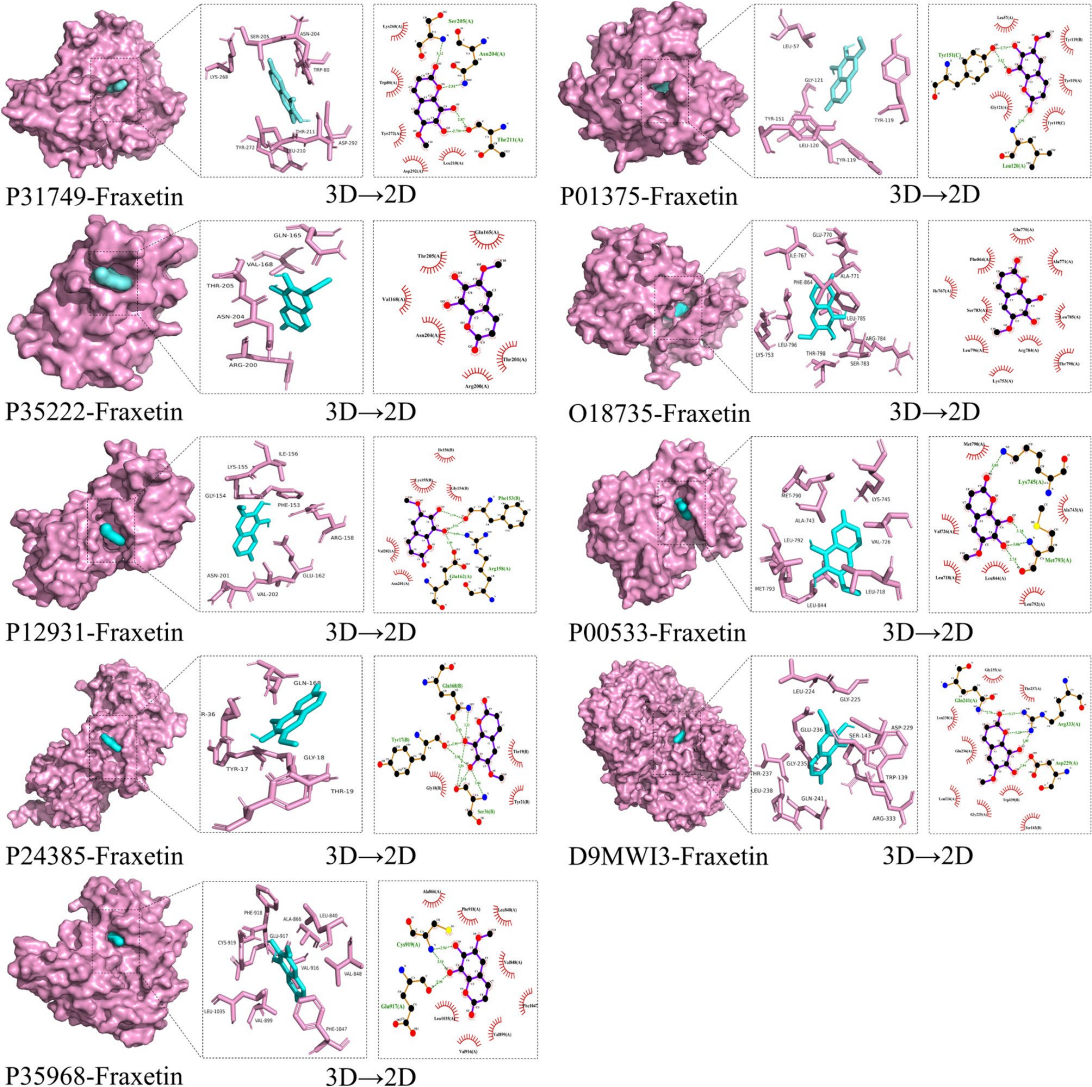
The molecular docking results. In 3D graph, the pink lump means hub target, blue lump means fraxetin. In bar graph, pink means amino acids, blue means fraxetin. In 2D graph, the green dotted means hydrogen bond

To further confirm the interaction between fraxetin and hub targets, we used fraxetin and hub targets as ligands and receptors for molecular docking analysis in AutoDock vina software. For each binding pair of ligands and receptors, we obtained 20 binding modes and the corresponding binding affinity (Table 2). A binding energy is less than − 5.0 kcal/mol or -7.0 kcal/mol [17] is thought to indicate that the binding activity between the ligand and receptor is better; the lower the binding energy, the higher the affinity between the receptor and the ligand, and the more stable the molecular conformation. Therefore, we chose the modes with the least binding energy using PyMOL and Ligplot software to evaluate the interaction between ligands and hub targets and map the 3D and 2D structure of the complex (Fig. 6). The results showed binding between fraxetin and the hub targets. Among the hub targets, AKT1, TNF and KDR have the strongest binding affinity with fraxetin, which are − 8.0, -8.3 and − 8.1 kcal•mol-1 respectively. Many previous studies have demonstrated the important role of AKT1, TNF and KDR in AML cell proliferation and chemotherapy resistance. The results of molecular docking showed that the anti-AML effect of fraxetin may be closely related to AKT1, TNF and KDR.

**Table 2:** Prediction of binding energy in Fraxetin against AML

PDB ID	Uniprot protein ID	binding affinity /kcal•mol-1
7nh5	P31749	-8.0
7kpa	P01375	-8.3
7afw	P35222	-5.7
7pcd	O18735	-6.9
1a08	P12931	-6.0
7vre	P00533	-6.7
2w9z	P24385	-5.9
5ikv	D9MWI3	-7.5
6xvk	P35968	-8.1

### Analysis of survival in relation to shared targets

**Fig. 7:**
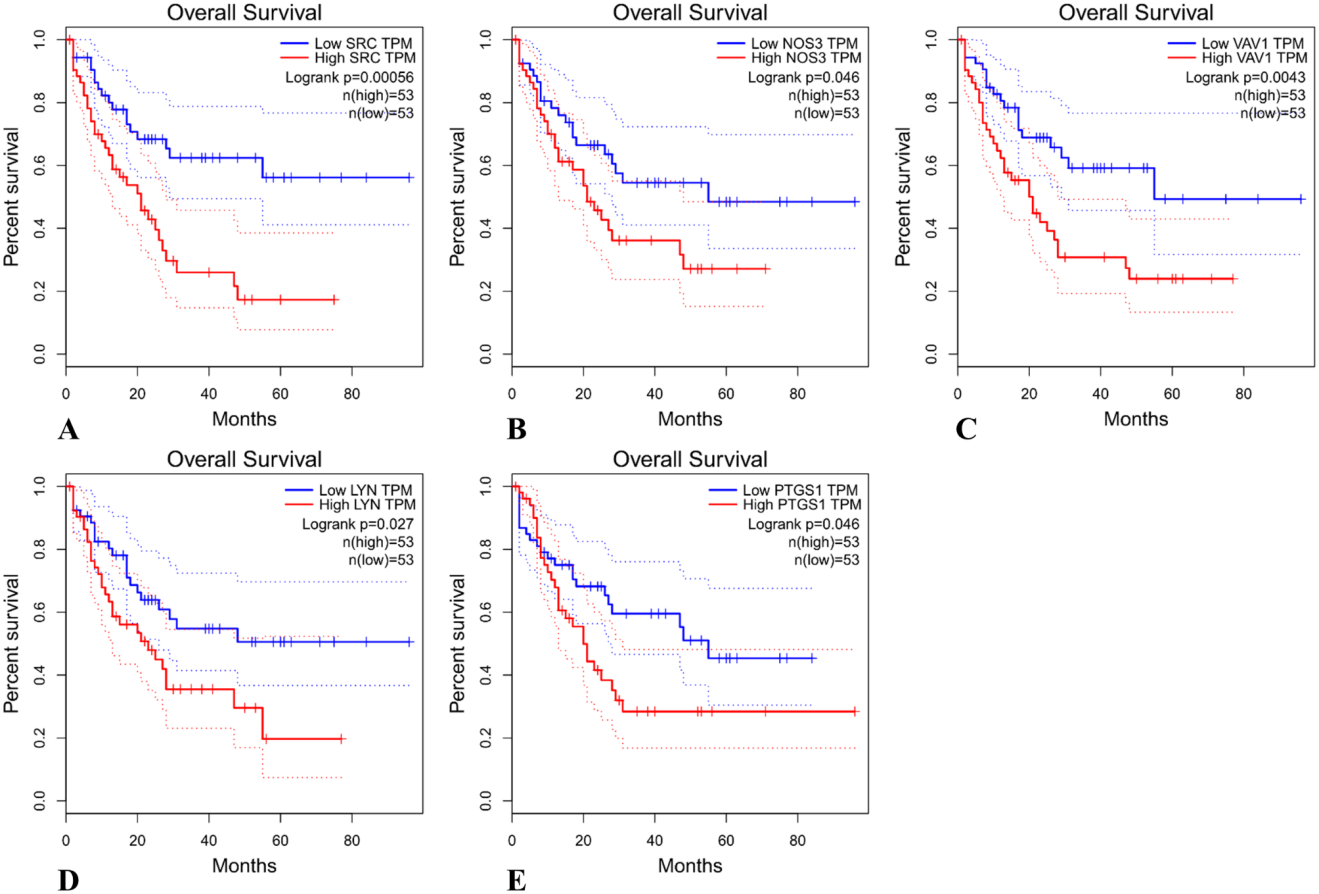
The overall survival analysis. A~E. The overall survival analysis of SRC, NOS3, VAV1, LYN and PTGS1(*p* < 0.05)

The analysis of survival in relation to shared targets in AML samples was performed using the GEPIA database, and the results showed that the expression of the shared targets SRC, NOS3, VAV1, LYN, and PTGS1 was significantly correlated with overall survival (p value < 0.05), and high expression of these targets indicated a poor prognosis for AML patients; the expression of the other shared targets was not significantly related to the overall survival of AML patients (Fig. 7). This result suggested that fraxetin may affect the prognosis of AML patients by regulating targets such as SRC and NOS3.

## Discussion

AML is a relatively common malignancy. According to the US National Institutes of Health (NIH) statistics, AML accounted for 1% of all newly diagnosed cancer cases in 2022, and its five-year survival rate is approximately 30.5% [18]. Although chemotherapy, stem cell transplantation, and other treatment options are becoming increasingly mature, many researchers are concerned about developing new drugs due to adverse reactions caused by chemotherapy drugs. Monomers of traditional herbal medicine have become the focus of attention of Chinese scholars. Although fraxetin has been reported to play a tumor suppressive role in a variety of human cancers, including breast, colon, and prostate cancers [7–9], no studies have reported its role in leukemia. This study is the first to suggest that fraxetin may have anti-AML effects.

Through the CCK-8 assay, we found that fraxetin can inhibit the proliferation of AML cells (Fig. 2A-B). The most common and well-defined type of programmed cell death is apoptosis, which is a common pathway for inducing cancer cell death [19]. In this study, through flow cytometry, we found that fraxetin can indeed induce apoptosis in AML cells, and early apoptosis was widely observed (Fig. 2C-E). These results revealed that fraxetin promoted the apoptosis of AML cells.

Network pharmacology can be used to analyze “ingredient-target-disease” networks to systematically determine the correlation between drugs and diseases and reveal the advantages of the multiple targets and pathways of active ingredients of traditional herbal medicine [20]. To further clarify the possible targets and pathways underlying the anti-AML effect of fraxetin, we used network pharmacology to construct biological action networks such as the “C-D-T and C-D-T-P networks”; we also performed GO function and KEGG enrichment analyses of these targets, further verified them through molecular docking, and tried to explore and analyze the potential targets and mechanism of action of fraxetin against AML.

First, we identified 29 potential targets of fraxetin in AML. Then, the “C-D-T and C-D-T-P” networks and PPI network showed that the hub targets of fraxetin against AML were AKT1, TNF, CTNNB1, ERBB2, SRC, EGFR, CCND1, PTGS2, and KDR. The molecular docking results showed that fraxetin could spontaneously interact with these hub targets (Fig. 6). In addition, the results of survival analysis showed that the high expression of SRC, NOS3, VAV1, LYN and PTGS1 was correlated with poor overall survival in AML patients (p value < 0.05) (Fig. 7). AKT is an effector downstream of PI3K in the PI3K/AKT signaling pathway and plays an irreplaceable role in the occurrence and development of AML [21]. AKT overexpression or the abnormal activation of phosphorylation can promote tumor development, resulting in resistance to traditional chemotherapy in patients; thus, AKT is a very attractive target for cancer treatment [22]. Zhou et al. found that curcumin can inhibit the phosphorylation of AKT in AML cells, thereby inducing apoptosis and inhibiting cell proliferation [23]. TNFs, including TNF-α and TNF-β, are central regulators of inflammation and have been linked to the occurrence and progression of many types of cancer, including AML; furthermore, TNFs can promote the production of leukemia stem cells [24]. In addition, upregulation of TNF-α expression can induce the accumulation of reactive oxygen species (ROS), leading to the apoptosis of AML stem cells [25]. In this study, we found that high TNF expression indicated a poor prognosis in AML patients. The β-catenin protein encoded by the CTNNB1 gene is a protein with a variety of biological functions in the Wnt signaling pathway, and a previous study showed that the Wnt/β-catenin signaling pathway was closely related to leukemia. Overexpression and translocation of the β-catenin protein in AML patients are often associated with poor prognosis in AML patients, and the protein is involved in the process of leukemia stem cell self-renewal; β-catenin may be a new therapeutic target for the elimination of leukemia stem cells [26, 27]. Overactivation of SRC family kinases (SFKs) is needed for the downstream signaling of membrane receptors in hematologic malignancy diseases, and many studies suggest that SFKs are reasonable therapeutic targets for AML [28]. The SRC-AKT signaling pathway is an indispensable signaling pathway in AML, and inhibition of SFK can reduce AKT phosphorylation and the expression of Mcl-1, induce the apoptosis of AML cells, and inhibit cell proliferation [29]. LYN is one of the main SFKs and is highly expressed and activated in AML cells, and knocking down LYN can affect the phosphorylation of mTOR targets and significantly inhibit the proliferation of AML cells [30]. In addition, we found that high expression of SRC and LYN was significantly associated with poor prognosis in AML patients. Abnormal expression of cyclin CCND1 (cyclin D1) is widely present in various tumors, and cyclin D1 helps the cell cycle enter the S phase, promotes cell proliferation, and participates in the regulation of cell metabolism and migration [31]. PTGS, also called cyclooxygenase (COX), is a rate-limiting enzyme in prostaglandin synthesis, and COX-1 activity has been shown to enhance the differentiation of all-trans-retinoic acid in acute promyelocytic leukemia [32]. Our survival analysis results showed that high PTGS1 expression was associated with poor prognosis in AML patients. The upregulation of COX-2 expression is closely related to the occurrence and progression of human cancer, and COX-2 inhibitors reduce the level of inflammatory factors, downregulate VEGF expression to inhibit tumor angiogenesis, and inhibit the PI3K-AKT signaling pathway to induce apoptosis in tumor cells [33]. A combination of COX-2 inhibitors and chemotherapy drugs can be used to reduce the toxic side effects of chemotherapy drugs, enhance the induction of apoptosis, reduce tumor angiogenesis, and improve the antitumor effect [34]. The COX-2 inhibitor celecoxib and its derivatives can induce the apoptosis of AML cells through the caspase-8-dependent apoptosis pathway by targeted inhibition of the focal adhesion signaling pathway [35]. VEGFR-2 (KDR) is a key signal sensor for physiological and pathological angiogenesis, and a previous study showed that VEGFR-2 can directly or indirectly promote the proliferation of HL-60 cells, revealing that VEGFR-2 can be a target for drug intervention, inhibit tumor cell proliferation and promote apoptosis [36]. Endothelial nitric oxide synthase 3 (NOS3) is involved in regulating the production of ROS, which plays an important antitumor role in AML patients and can stimulate leukemia-related apoptotic pathways through oxidative stress, DNA damage, membrane damage, and lipid peroxidation; NOS3 is correlated with the overall survival of AML patients [37], and this finding is consistent with our survival analysis. VAV family genes (VAVs), which are located downstream of protein tyrosine kinases, including VAV1, VAV2, and VAV3, are signal transduction molecules that are regulated by tyrosine phosphorylation and are associated with the occurrence, progression, and prognosis of many cancers [38]. VAV1 is mainly expressed in hematopoietic cells, and the complete response rate of AML patients with high VAV1 expression is lower than that of AML patients with low VAV1 expression; high VAV1 expression is also associated with poor overall survival, suggesting that high VAV1 expression is associated with poor prognosis in AML patients [39]. In summary, fraxetin may exert anti-AML effects by interacting with the above targets to produce a variety of biological effects, such as inhibiting cell proliferation and inducing apoptosis, as well as reduce the toxic side effects of chemotherapy drugs and affect the prognosis of AML patients.

To explore the potential anti-AML mechanism of fraxetin, we subsequently performed enrichment analysis. The results showed that the possible biological pathways of fraxetin against AML mainly included oxidative stress, protein autophosphorylation, and cellular response to drugs, and fraxetin may act on multiple pathways, such as focal adhesion and the PI3K-AKT signaling pathway. The focal adhesion signaling pathway is initiated by integrin activation, which contributes to almost every aspect of cancer cell activity, and activation of this pathway leads to downstream cascade activation, including activation of the PI3K-AKT signaling pathway, which promotes tumor survival and development [40]. Most gene mutations in AML can lead to the excessive activation of integrins and regulation of the focal adhesion signaling pathway; furthermore, the aforementioned COX-2 inhibitor celecoxib and its derivatives can induce apoptosis in AML cells through the caspase-8-dependent apoptosis pathway by targeting the focal adhesion signaling pathway. The PI3K-AKT signaling pathway can inhibit apoptosis, and this pathway is overactivated after stimulation by certain biological factors; overactivation of this pathway promotes cell proliferation and migration and inhibits apoptosis [41]. Moreover, many recent studies have shown that different kinds of traditional herbal medicine or their active ingredients can inhibit the proliferation of various tumor cells, including AML cells, and induce apoptosis by inhibiting PI3K-AKT signaling [42–44]. Therefore, we speculated that fraxetin may play an anti-AML role by regulating focal adhesion and the PI3K-AKT signaling pathway.

In view of the current unsatisfactory effects of AML treatments, new therapeutic strategies are still urgently needed. In this study, we provide several potential targets for AML therapy, which may facilitate the development of new therapeutic strategies. In recent years, the effect of fraxetin in the treatment of tumors has proven satisfactory. However, its application in AML is rarely studied. In this paper, we preliminarily explored the potential molecular mechanism of fraxetin in the treatment of AML based on network pharmacology and molecular docking. However, this study has some limitations. First, as data from online databases are based on assessments, it is possible that undocumented or unvalidated chemicals or targets were not included in our study. Second, conducting a more in-depth study on the metabolic form, active ingredients, and absorption mechanism of fraxetin would be ideal. Further in vitro and in vivo studies are needed to explore the mechanism of action of fraxetin in the treatment of AML.

## Conclusion

In summary, in this study, we evaluated the effects of fraxetin on the proliferation and apoptosis of AML cells through cell experiments. The potential targets and signaling pathways of the anti-AML effects of fraxetin were predicted by network pharmacology. Through molecular docking experiments, we confirmed that fraxetin had good binding activity with hub targets. In addition, the correlation between overlapping targets and overall survival in AML patients was analyzed. This study provides ideas for further experimental research and the clinical application of fraxetin.

### Electronic supplementary material

Below is the link to the electronic supplementary material.

Supplementary Material 1

Supplementary Material 2

## Data Availability

The datasets used and analyzed during the current study are available from the corresponding author on reasonable request.

## Abbreviations

AML: Acute myeloid leukemia
PPI: Protein-protein interaction
CCK-8 Cell: Counting Kit-8
C-D-T: Compound-Disease-Targets
PPI: Protein-protein interaction
GO: Gene Ontology
KEGG: Kyoto Encyclopedia of Genes and Genomes
C-D-T-P: Compound-Disease-Targets-Pathway
BP: Biological Processes
CC: Cellular Components
MF: Molecular Functions
