# Supplementary figures and images for: Exploring the mechanism of fraxetin against acute myeloid leukemia through cell experiments and network pharmacology
Source: BMC Complement Med Ther. 2024 Jun 10;24:226. doi: 10.1186/s12906-024-04529-8 (PMC11163689; doi:10.1186/s12906-024-04529-8)

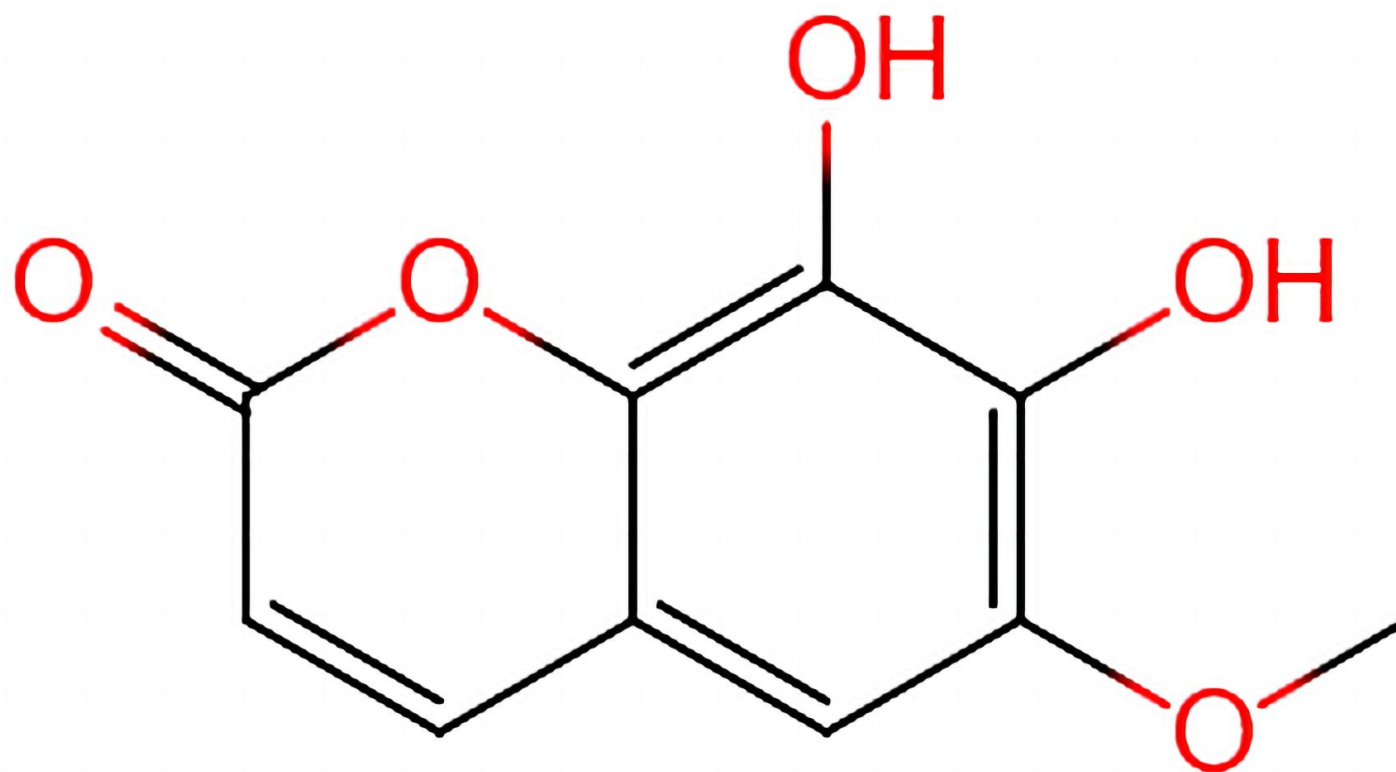

Figure S1:Chemical Structure of Fraxetin.

Supplement: Supplementary file 1 — Supplementary Material 1 [file 12906_2024_4529_MOESM1_ESM.pdf]
