# Supplementary material for: Exploring the mechanism of fraxetin against acute myeloid leukemia through cell experiments and network pharmacology
Source: BMC Complement Med Ther. 2024 Jun 10;24:226. doi: 10.1186/s12906-024-04529-8 (PMC11163689; doi:10.1186/s12906-024-04529-8)
Supplement: Supplementary file 2 — Supplementary Material 2 [file 12906_2024_4529_MOESM2_ESM.pdf]

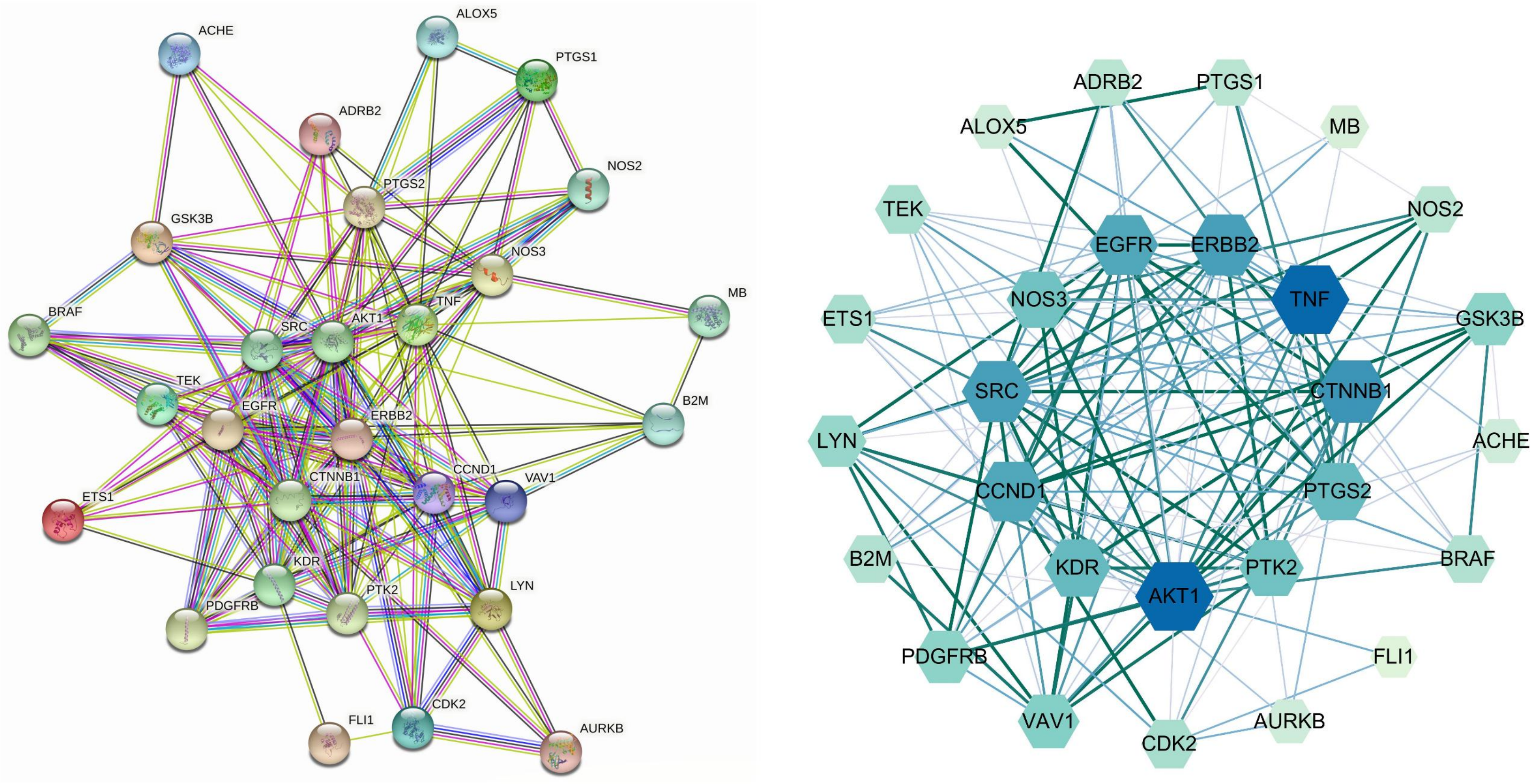

A B

Figure S2:The protein-protein interaction (PPI) network and hub targets screening. A. PPI network: nodes represent proteins, edges represent protein-protein associations. B. Hub targets screening: The edges from thin to thick indicate the combined score changes from small to large.
